# Noncontact Laser Ultrasound Detection of Cracks Using Hydrophone

**DOI:** 10.3390/s21103371

**Published:** 2021-05-12

**Authors:** Ki Chang Kang, Kwan Kyu Park

**Affiliations:** Department of Mechanical Convergence Engineering, Hanyang University, Seoul 04736, Korea; kkchzzang@hanyang.ac.kr

**Keywords:** laser ultrasonic, crack detection, impedance mismatch, noncontact NDE

## Abstract

We present a noncontact, non-immersion ultrasonic inspection method. A broadband ultrasound signal generated by a pulsed laser was measured using a hydrophone. The generated ultrasound signals propagated through the specimen and received a signal from the hydrophone in the water. Soldered chip ceramic capacitors, resistors, and surface-mount-type chip amplifiers were used as experimental specimens. A polydimethylsiloxane layer was used to prevent the specimen from being impacted by contact with water. The presence of a crack in the middle of the specimen resulted in an air layer, and the intermediate air layer reduced the magnitude of the signal transmitted owing to impedance mismatch. Using this principle, the cracks in each specimen could be distinguished. The image contrast ratio derived from the proposed method is approximately two to three times higher than that derived using the conventional immersion ultrasonic method. These results show that the proposed method can replace existing immersion-type ultrasound transmitted images.

## 1. Introduction

Nondestructive testing (NDT) is extensively used in various fields, such as mechanical and civil engineering and aerospace systems. There is an increasing demand for ultrasonic techniques that can detect small defects and cover a large area [1,2,3,4,5]. The most common method is the contact-type transducer method, based on one or several transducers in contact with the subject under inspection [6,7,8,9,10]. However, this method requires contact or embedded transducers and has several disadvantages. It is often difficult to obtain a sufficiently high spatial resolution to detect small initial damage as the transmission and receiving signals operate on discrete points. In addition, the price and complexity associated with the cable increase with the number of contact-type transducers. Many contact-type transducers do not work in certain environments, such as high-temperature and radioactive conditions, and can change the dynamic characteristics of the target structure. To solve these problems, the need for noncontact laser ultrasound technology has increased.

Typically, there are two types of noncontact ultrasonic techniques: water immersion ultrasonic [11,12,13,14,15,16] and noncontact laser ultrasonic. The water immersion ultrasonic technique is mostly performed on small objects with uneven surfaces. Here, water is used as the coupling medium, and a reflective or transmission signal can be acquired and used to detect defects. Because of the good coupling, high-frequency (<50 MHz) ultrasound detects cracks inside the object with high resolution. However, the immersion technique inevitably requires a wet and dry process and is not preferred in many industrial fields. By contrast, noncontact laser ultrasonic excitation is generally performed using a pulsed laser [17,18,19,20,21]. Ultrasound generated from pulsed light propagates through an object, and defects can be detected from the measured wave propagation. Wave propagation is typically measured by a laser interferometer, which is the most representative noncontact vibration measurement method [21,22,23,24].

Another method uses local resonances of the structure that appear at frequencies at which the group velocity of some modes vanishes with a laser interferometer [25]. Using noncontact laser ultrasonic technology, it is possible to construct an ultrasonic object image with high spatial resolution without a specific sensor arrangement. This has the advantage of detecting the damage without reference data regarding the initial condition of the object. Moreover, the object is less vulnerable to malfunction owing to changes in the environment and operating conditions, and there is a reduction in the number of sensors and cables.

However, there are some limitations to the noncontact ultrasonic technique. The quality of the measured ultrasound signal depends specifically on the condition of the object surface and the incident angle of the laser beam [26]. In addition, when using a laser interferometer, owing to the complexity and cost problems of optical beam steering systems, there are disadvantages such as galvanometers and other expensive equipment. Because these ultrasonic techniques are based on Lamb waves that propagate in platelike structures, they have been mainly used for plane surfaces, and they are difficult to implement on complicated surfaces, such as assembled printed circuit boards (PCBs).

In this study, to advance conventional methods, we propose a noncontact laser ultrasound system based on a pulsed laser and hydrophone. An ultrasound signal was generated by a pulsed laser, and the transmitted signal was acquired by a hydrophone during immersion. To use the hydrophone in water, a polydimethylsiloxane (PDMS) layer was used to protect the test object from water while also acting as a coupling layer. The proposed noncontact laser ultrasonic technique shows a high level of performance compared with the conventional water immersion ultrasonic method.

The remainder of this paper is organized as follows: Section 2 describes the equipment arrangement and specimen used in the experiment. The experimental results and a comparison with the conventional water immersion ultrasonic method are explained in Section 3. The conclusions are presented in Section 4.

## 2. Principle and Simulation

### 2.1. Principle of Noncontact Laser Ultrasonic System

**Figure 1:**
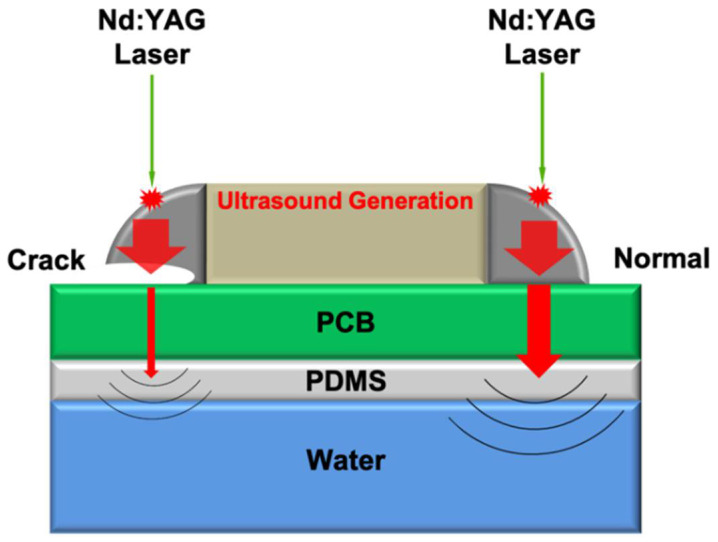
Principle of noncontact laser ultrasonic system.

The proposed noncontact laser ultrasonic method uses a pulsed laser and a hydrophone to measure the acoustic signal. The generated ultrasound signals propagate through the specimen, and the amplitude of the passing signals differs depending on the presence or absence of a crack. A crack causes more reflection of the transmitted ultrasound signal owing to the acoustic impedance mismatch (Figure 1). Thus, the presence or absence of a crack is determined using the magnitude of the received signal.

The transmitted ultrasound signal propagates through the specimen, and the penetrated signal is measured by a hydrophone in water. The ultrasound signal causes internal reflection and interference when it propagates through the specimen. Therefore, the initial part of the received signal is used to reconstruct the image and detect the defects. Unlike the conventional laser ultrasound technique using a laser Doppler vibrometer (LDV), the proposed method is not affected by the specimen surface or the reflectivity of light. In addition, it is suitable for various objects, including multiple layers.

### 2.2. Finite Element Analysis

**Figure 2:**
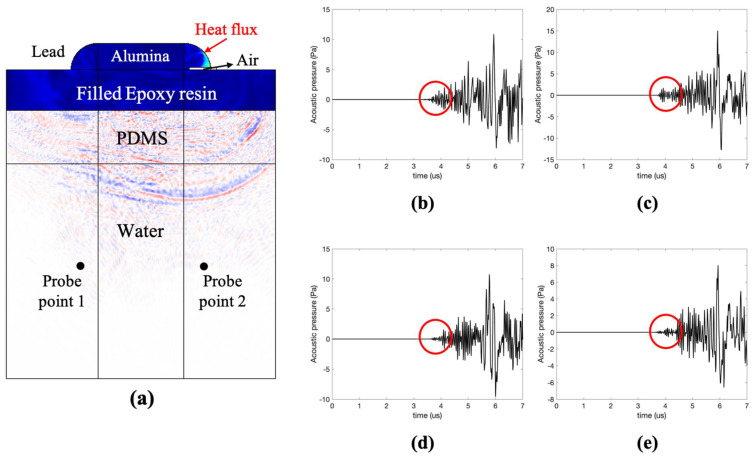
(**a**) The geometry of the simulation model, and wave propagation of the generated acoustic signal at cracked part, A-scan data of (**b**) normal part, (**c**) 0.1 mm-wide crack, (**d**) 0.5 mm-wide crack, and (**e**) 1 mm-wide crack.

To validate the proposed method, finite element analysis (FEA) was conducted under conditions similar to the experimental conditions. FEA was conducted using a commercial FEA package (COMSOL Multiphysics, COMSOL, Inc., Burlington, MA, USA). To implement the FEA under the same conditions as the experiments, the cross-section geometry is based on the experiments, as shown in Figure 2a. The chip ceramic part (resistor) consists of a rectangle of 3.2 mm × 1 mm and is modeled as made of alumina. The PCB and PDMS parts consist of a rectangle with a width of 1 cm and height of 1.5 mm and 2 mm, respectively. The speed of sound, density, Young’s modulus, and Poisson’s ratio of PDMS is 990 m/s, 970 kg/m^3^, 0.75 GPa, and 0.4, respectively. These parameters of PDMS are the same as the ones used in experiments. The PCB part was modeled as a filled epoxy resin. The lead has a circular shape, and the air gap is placed with a thickness of 0.1 mm. The material properties of the alumina-filled epoxy resin and the lead for the heat transfer model used in the FEA are listed in Table 1.

The crack widths were set to 0.1 mm, 0.5, and 1 mm to examine their effects. The pulse laser irradiation was imitated by the heat flux (general inward heat flux) of 7 ns on the solder surface, calculated based on the pulsed laser used in the experiment. The thermal expansion of lead generates the solid displacement, and the acoustic data were acquired at the water. The lead part was made of small mesh size for the accurate performance of the thermal expansion in the early stage of the simulations. The ultrasound wavelength of water at 3.3 MHz is 454 μm. Based on the wavelength at the frequency of interest, the maximum element size of the lead part and the remaining part is 22 μm (λ/20) and 45 μm (λ/10), respectively. The acoustic pressure was transmitted to the PCB through the solder, and most of the acoustic pressure at the defective part was reflected in the air layer by the acoustic impedance mismatch. The probe points were aligned with the excitation location. The pressure data from the soldered part acquired at probe point 1 are shown in Figure 2b. The pressure data from the defective soldered part acquired at probe point 2 at 0.1 mm, 0.5, and 1 mm are shown in Figure 2c–e.

Notably, the initial signal excludes internal reflection. To compare the effect of the cracks, the maximum pressure and peak-to-peak pressure were calculated from 3.6 μs to 4.2 μs. In the case of a 0.1 mm-wide crack, which is smaller than the laser spot size and half the wavelength, the defective soldered signal had a 19% lower amplitude than the normal soldered signal, as shown in Table 2.

## 3. Experimental Setup

**Figure 3:**
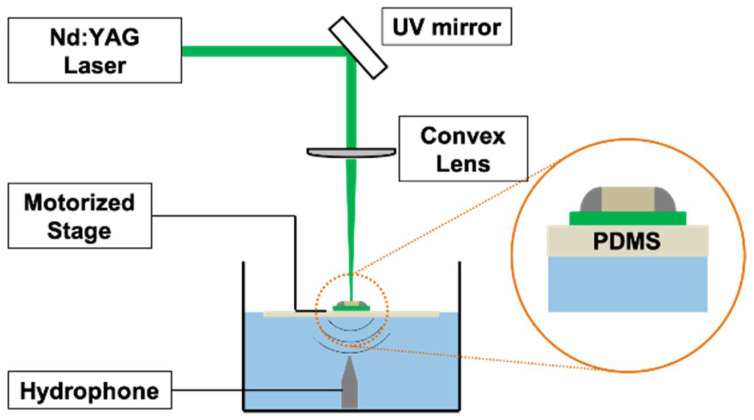
Schematic of noncontact laser ultrasonic system.

A schematic of the laser ultrasound system used in the proposed method is shown in Figure 3. The Nd:YAG laser (Minilite I, Amplitude, San Francisco, CA, USA) has a wavelength of 532 nm, a pulse duration of 7 ns, a pulse repetition frequency of 10 Hz, and a maximum energy of 27 mJ with a spot diameter of 3 mm. A UV mirror and convex lens were used to irradiate and focus the laser on the specimen. By focusing the laser, it is possible to increase the amplitude of the ultrasonic signal passing through the specimen because the same pulse laser energy provides concentrated energy and increases the generated signal. In addition, a smaller spot size improves the resolution of the crack-detection system. However, when the laser is irradiated into a very small spot size, the energy emitted from the laser is concentrated in a narrow area, which can damage the specimen.

In the experiments, the spot diameter was set to be 0.2 mm by considering the resolution and concentrated energy of 6 mJ per pulse. The ultrasound signal propagated through the specimen was received by a broadband hydrophone (HNP-0400, ONDA Corp., Sunnyvale, CA, USA) with a frequency range of 1–20 MHz. The data were acquired with an average of eight repetitions using an oscilloscope with an amplifier with a gain of 40 dB. Because the laser generates a broadband signal, signal measurement was conducted using a wideband hydrophone, which increased its applicability to various objects. The object was a soldered PCB with a normal soldering part and a cracked part. The specimen was placed on a 2 mm-thick PDMS (Sylgard 184, Dow Corning Co., Midland, MI, USA) layer, and half the PDMS was immersed in water. The PDMS layer accomplished two roles: it acted as a coupling material that minimized the acoustic impedance mismatch, and at the same time, allowed the object to be immersed in water, enabling using a hydrophone. The object and the PDMS layer were connected to a motorized stage (SM3-0820-4S, Sciencetown Inc., Incheon, Korea) for precise movement and area scanning. For sample A, the scanning area was 6 mm × 6 mm, and scanning was performed by moving 100 μm in the x and y directions. For sample B, the scanning area was 10 mm × 6 mm, and scanning was performed by moving 250 μm in the x- and y-directions.

**Figure 4:**
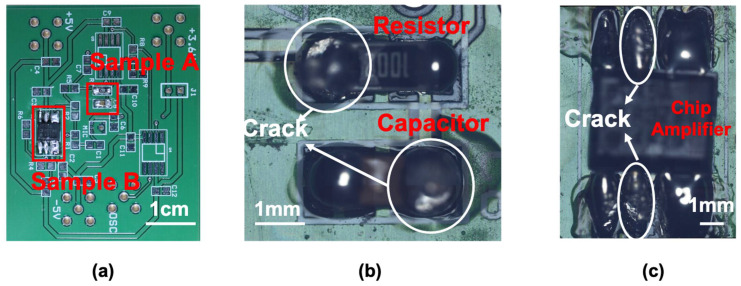
Specimen: (**b**) sample A (resistor and capacitor) and (**c**) sample B (AD8045 chip amplifier).

The experiments were conducted using two parts of the specimen for application under various conditions. Sample A consisted of a 1 mm-thick chip ceramic resistor and 1 mm-thick chip ceramic capacitor with a 1206 size (3.2 mm × 1.6 mm), as shown in Figure 4b. Sample B was a commercial surface-mount-type chip amplifier (AD8034, Analog Devices Inc., Norwood, MA, USA) soldered to the PCB, as shown in Figure 4c. Sample A had a crack on the left side of the resistor and a normal solder on the right side of the resistor. In the case of the capacitor in sample A, a crack was present on the right side and a normal solder on the left side. The chip amplifier of sample B had eight legs: the upper legs soldered normally, and the lower two legs soldered together. Both sides of the middle legs had cracks, as shown in Figure 4c. The crack size of sample A was approximately 0.8 mm, and the crack size of sample B was approximately 0.5 mm.

## 4. Experimental Results

### 4.1. Experimental Results: Raw Data

**Figure 5:**
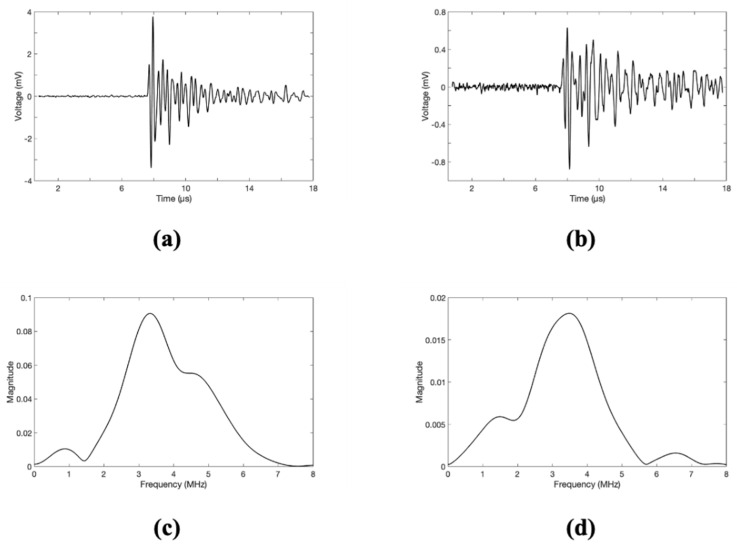
Measured acoustic signal through a resistor (**a**) at the center of normal soldering part, (**b**) at the center of cracked soldering part, FFT result (**c**) at the center of soldering part, and (**d**) at the center of cracked soldering part.

Experiments based on the proposed method were conducted using the specimens mentioned above, and the results from sample A are shown in Figure 5. The maximum penetrated signal of a normal soldering part has a magnitude ranging from 2.5 mV to 4 mV, as shown in Figure 5a. However, the penetrated signal of the cracked part has a magnitude of 0.6 to 1.2 mV, as shown in Figure 5b. In the case of the cracked part, the reflection by the acoustic impedance mismatch occurred because of the intermediate air layer, and the amplitude of the penetrated signal was reduced by 2–4 times that of the normal part. The frequency analysis results by the fast-Fourier transform (FFT) indicate that the center frequency formed at approximately 3.3 MHz, as shown in Figure 5c,d.

The magnitude of the FFT results of the normal part was high because the ICR of the signal was better, and the signal amplitude was high. The center frequency of the received signal was not affected by the presence of cracks. The motorized stage moved only in the x- and y-axes, and the z-axis was fixed during scanning. The first arrival time of the penetrated signal was measured constantly at approximately 10 μs regardless of the crack. As the time zone after the first arrival time includes multipath signals, including internal reflections, it is not suitable for analysis using the proposed method. Therefore, C-mode imaging for detecting cracks was reconstructed using a time zone of approximately 10 μs of the penetrated signal.

### 4.2. Scanning Results

Before reconstructing the C-mode image, the measured signal was processed using a bandpass filter with a center frequency of 3.3 MHz to reduce undesirable noise effects.

**Figure 6:**
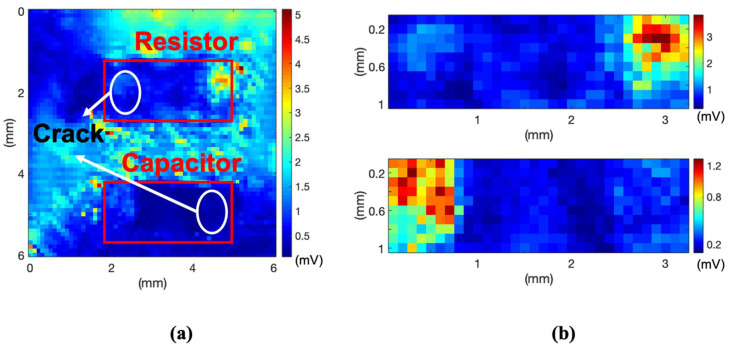
(**a**) C-mode image and (**b**) partial image of red rectangle area.

As mentioned above, the C-mode image was reconstructed using the signal at approximately 10 μs because this time zone has proper information about the signal magnitude reduction owing to the acoustic impedance mismatch. The C-mode image of sample A is shown in Figure 6a. The part marked with a red rectangle is the location of the soldered package with a chip ceramic resistor and capacitor. The chip ceramic resistor has a crack on the left side of the device, and the capacitor has a crack on the right side. For the entire scanned area, the laser ultrasonic signal from the single PCB, which has no devices, has a higher amplitude than the part, including the PCB with solder. To clarify and distinguish the crack, the area measured, selected, and imaged is indicated by the red rectangle, as shown in Figure 6b.

In the case of the resistor, the left part with a crack shows a smaller amplitude than the right normal soldering part. In the case of the capacitor, like the results of the resistor, the signal on the left side, which is normally soldered, is higher than on the right side where the crack exists. For sample A, the middle part of the chip ceramic device and the contact between the device and PCB are very weak. As soldering was conducted only on both sides of the device and not the middle part, the middle part had a thin air layer. Therefore, the measured signal at the middle part of the device was similar to the previously cracked parts but smaller than the normal soldering part. However, this is not an issue. The purpose of this experiment was to distinguish the cracked part of the soldering. In other words, it is appropriate to use this method under the same conditions except for the cracked part.

**Figure 7:**
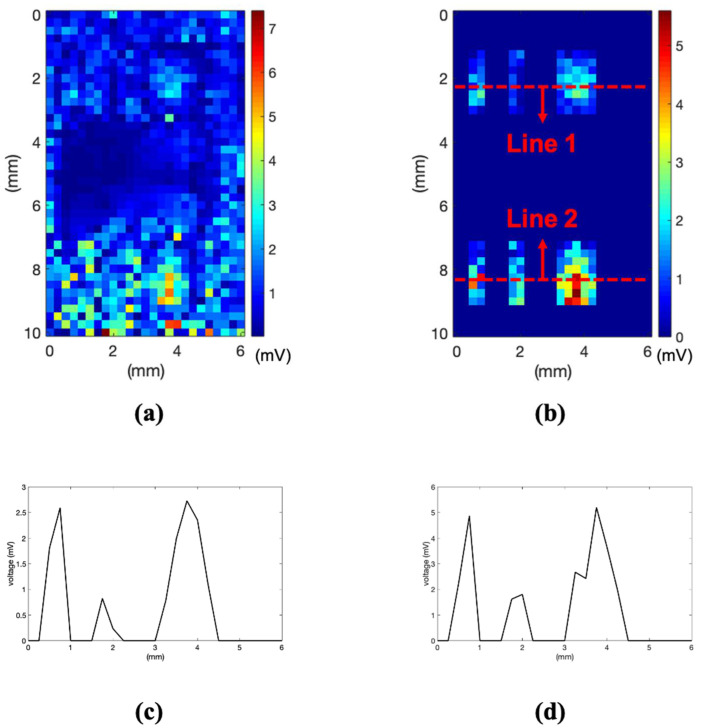
(**a**) C-mode image, (**b**) image reconstructed with only values of soldering part, (**c**) received voltage value along line 1, and (**d**) received voltage value along line 2.

Considering the laser diameter to be 0.2 mm, experiments on sample B were conducted to check the possibility of detecting smaller cracks. Sample B, a soldered surface-mount-type chip amplifier with a leg thickness of 0.5 mm, was measured using a noncontact laser ultrasonic method. The entire C-mode image reconstructed for sample B is shown in Figure 7a. Owing to the high amplitude of the penetrated signal from the single PCB part, it is difficult to distinguish between the normal and cracked parts.

The image result obtained using only the signal from the soldering part of the chip amplifier leg is shown in Figure 7b, which is a partial image for comparing the same specimen component. The main idea of this technology is detecting the crack in a designated area, which is in the soldering part of the experiment. The only prior knowledge used in the experiment is the location of the soldering part on the PCB based on the layout. The partial image was reconstructed based on the coordinates of the layout. To accurately distinguish the normal soldering parts of the single leg and two legs and the cracked part, the plotting results along lines 1 and 2 are shown in Figure 7c,d. These results show that the maximum amplitude of the normal part is two to three times higher than that of the cracked part. The proposed laser ultrasonic method has a resolution of less than 0.5 mm from the experiment results.

### 4.3. Comparison with Conventional Water Immersion Ultrasonic Method

**Figure 8:**
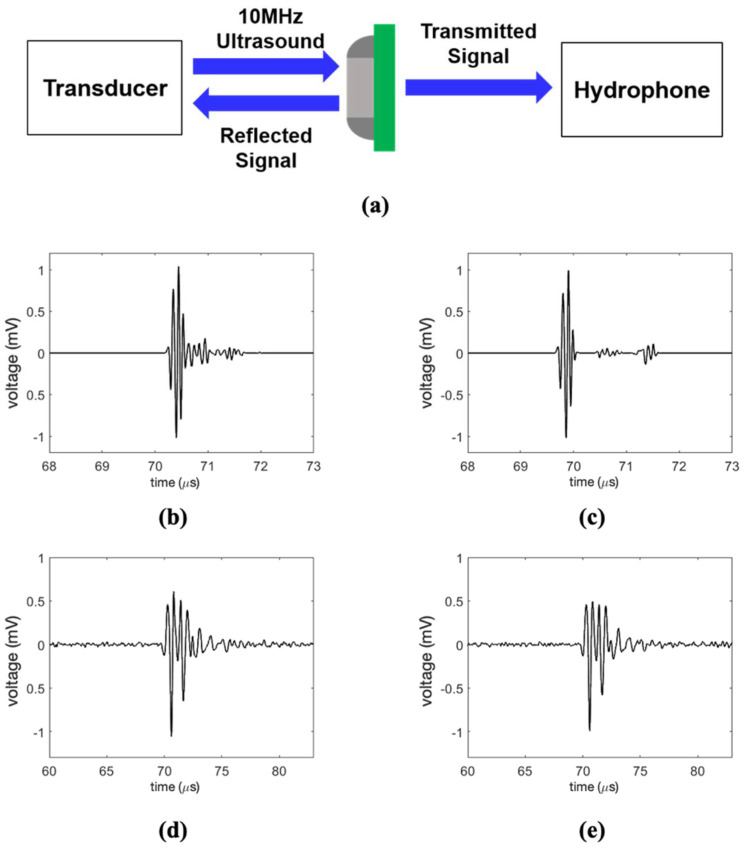
(**a**) Schematic of existing immersed ultrasonic method, reflected signal acquired by the transducer (**b**) from normal part, (**c**) from cracked part, penetrated signal acquired by hydrophone (**d**) from normal part, and (**e**) from cracked part.

To compare the proposed method with the conventional water immersion ultrasonic method, the experiments with sample A were repeated, as shown in Figure 8a. A 10-MHz commercial focused ultrasound transducer (A321S-SU, Olympus Inc., Tokyo, Japan) with a 50.8 mm focal length, 19 mm diameter, and f-number of 2.67 was placed on one side; a hydrophone, which was the same as in the previous experiments, was placed on the other side. The ultrasound signal was generated and received by a pulser-receiver (5072PR, Olympus Inc., Tokyo, Japan), and the transmitted ultrasound was reflected and penetrated the specimen. The reflected signal was received by a focused ultrasound transducer, and the penetrated signal was received by the hydrophone. This implies that the results of the transducer pulse–echo experiment and the hydrophone transmission signal test were simultaneously measured.

**Figure 9:**
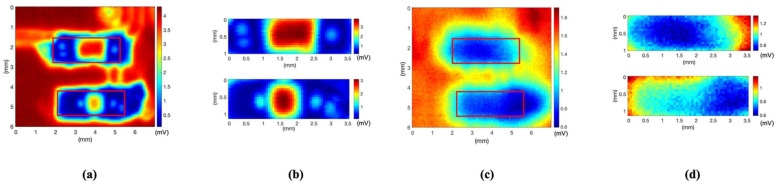
(**a**) C-mode image of transducer pulse–echo, (**b**) partial image of red rectangle area in transducer pulse–echo image, (**c**) C-mode image of the transmitted signal using a hydrophone, and (**d**) partial image of red rectangle area in hydrophone test.

The experimental results were acquired for the same area by conducting two experiments simultaneously. To match the focal point, the distance between the specimen and the transducer was fixed at 5.2 cm, and the area was scanned 6 cm × 6 cm while moving in 100-μm steps. The reflected signal from the normal part acquired by the ultrasound transducer is shown in Figure 8b. The reflected signal from the cracked part is shown in Figure 8c, and there is almost no difference between the normal and cracked parts. In the case of the penetrated signal acquired by the hydrophone, the penetrated signals from the normal and cracked parts are shown in Figure 8d,e, respectively. This shows the difference between the normal part and the cracked part, but the difference is smaller than in our proposed method. The result of the reflected signal through a pulse–echo experiment using a focused ultrasound transducer is shown in Figure 9a. C-mode imaging was reconstructed using the amplitude of the measured signal at the first signal arrival time zone.

The existing ultrasonic pulse–echo method uses the reflected signal from the target specimen, unlike the analysis of the penetrated signal used in the noncontact laser method suggested in this study. The reflected signal caused by the acoustic impedance mismatch occurs more at the cracked part, so the amplitude of the reflected signal from the normal part is smaller than that of the cracked part. Therefore, in the conventional pulse–echo method, the reflected signal from the cracked part has a higher amplitude in the C-mode image.

A partial image of the area marked with a red rectangle is shown in Figure 9b. However, the presence or absence of a crack even in the partial image, which excludes the single PCB parts, cannot be ascertained owing to the lack of difference. These results show that the conventional immersion pulse–echo method using a focused transducer is not suitable for distinguishing the crack of the specimen with a complex geometry surface. The C-mode image reconstructed by the penetrated signal received using a hydrophone is shown in Figure 9c. In this method, the principle used in the experiment is the same as that in the method proposed in this research because the penetrated signal is used to detect cracks.

In the case of the ultrasound experiments in water, the amplitude of the ultrasound penetrating through the crack was higher than that for the test in air. This is because the acoustic impedance difference between the specimen and water was smaller than between the specimen and air. Likewise, the partial image is indicated by the red rectangle without a single PCB is shown in Figure 9d. Compared with the noncontact laser ultrasonic image proposed in this study, cracks are not distinguishable because the acoustic impedance mismatch between the specimen and water is small. To compare the conventional water immersion ultrasound method and the method proposed in this paper, the image contrast ratio (ICR) was calculated.

(1) $$ICR=\frac{\mu_{normal}}{\mu_{crack}}.$$

Using Equation (1), the image contrast ratio was calculated by dividing the average of the normal partial signals by the average number of cracks, where the $\mu_{normal}$ is the average of the normal partial signals and $\mu_{crack}$ is the average of the cracked partial signals.

Table 3 shows the average values and ICR of the normal part and the crack part signal for the water immersion ultrasound method and the proposed laser ultrasonic method. The average value of the normal part and the crack part was calculated by averaging the maximum signal value of each point by selecting 25 points. In the case of the resistor, the ICR of the proposed laser ultrasonic method is 2.64 times better than that of the water immersion ultrasonic method. Similarly, in the case of the capacitor, the ICR of the proposed laser ultrasonic method is 2.35 times better than that of the water immersion ultrasonic method.

This demonstrates that the proposed laser ultrasonic method has better ICR and superior crack discrimination than the water immersion ultrasonic method. In our applications, the crack was connected to the medium such that the crack consisted of the medium. In this case, the medium is air. This means that ICR is related to the medium in this application. Therefore, the experimental results show that the proposed laser ultrasonic method performs better than the water immersion ultrasonic method for various defect cases. In addition, in an isolated crack, the ICR is determined by the internal medium. In most cases, the medium is filled with air, so the ICR is higher than the conventional immersion ultrasonic method.

## 5. Discussion

The conventional laser ultrasonic or air-coupled transducer (ACT) method is difficult to detect in the case of complex surface specimens, such as PCBs. LDV is not suitable for complex surfaces, and ACT has a low frequency, which results in low resolution. In particular, for the mode conversion case, ACT requires a specific angle for the specimen surface, which is a critical part of the complex surface. The most important factor in our proposed method is its high applicability for various surface conditions with high frequencies.

This method aims to measure the signal that passes through the specimen and changes in the amplitude of the first-arriving signal due to the crack and the intermediate air layer. However, this principle requires the same conditions to compare the presence or absence of cracks. In our experiment, the specimen has three parts: a single PCB part, a soldering part, and a chip on the PCB part. The crack exists in the soldering part, so the other parts with different original signals are unnecessary for detecting cracks. Therefore, complicated specimens require section separation to improve the clarity of the defect.

Using the intermediate coupling layer, the attachment between the layer and specimen is an important factor. In the case of any local separation or disbonding between them, there is the possibility of misinterpretation as a crack in the specimens. The PDMS was selected because of some characteristics, which are beneficial to the proper attachment. First, PDMS is known for its unusual rheological properties so that it is easy to attach to a flat surface with a neglectable air gap. Second, the PDMS is optically transparent in general. Thus it is also possible to check visually in the case of a sufficiently big air gap. In our case, the amplitude of the penetrated signal depended on the type of specimen component. There is no identifiable effect of dis-bonding between the PDMS layer and the specimen. In the case of the specimen with a complex bottom surface, the PDMS could be made with the shape of the bottom surface because it is easy to mold.

In addition, the center frequency of the generated acoustic signal from the pulsed laser is 3.3 MHz, but for the comparison experiments, a 10-MHz transducer was used because it is one of the most common frequencies of immersion techniques to detect cracks. Using a 10-MHz transducer, a high-resolution with almost the same contrast ratio could be obtained because the impedance mismatch is not affected by the frequency. Therefore, the proposed method is more suitable for complex specimens than the conventional immersion method.

The lateral resolution of the C-mode image obtained using the proposed method is related to the wavelength and laser spot size. The ultrasound wavelength of water at 3.3 MHz is 454 μm, half of the wavelength is approximately 227 μm, and the laser spot size is approximately 200 μm. Our case was conducted with a soldering sample; the lateral resolution was approximately 230 μm.

Because the present technique is a feasibility test using a single hydrophone, it requires a long scanning time. Depending on the scanning area, the reconstruction image of sample A consists of 3721 scanning points, and the reconstruction image of sample B consists of 1025 scanning points, which takes 1.5 s per point. The total scanning times take 1.5 h and 0.42 h in the current system, respectively. Most of the scanning time was owing to mechanical movement and averaging time. The measured data were averaged over eight repetitions to reduce the effects of vibration from the motorized stage. The fabrication of an ultrasound sensor array and using a pulse laser with a high repetition frequency can dramatically reduce the scanning time. In addition, in our experiments, the UV mirror, which does not match with a 532 nm laser, was used. Using the optical fiber or mirror, which matched wavelength with the pulse laser, could make it better efficient.

## 6. Conclusions

This paper proposes a convenient noncontact laser ultrasound method to detect small cracks. The proposed method was implemented using a pulsed laser and broadband hydrophone. The proposed solution avoids the complexity and cost of the conventional laser ultrasonic method using a laser interferometer and hydrophone. In addition, we addressed several problems owing to the hydrophone by adding a PDMS layer. The acoustic signal generated by a pulse laser penetrated through the specimen, and the hydrophone in the water was measured to determine the presence or absence of cracks.

If the crack part is the air layer, the maximum amplitude of the normal part was 2–3 times higher than that of the crack part. The performance improved compared with the conventional water immersion ultrasonic method despite the specimen not being immersed. The ICR of the proposed laser ultrasonic method was 2.35–2.64 times better than that of the water immersion ultrasonic method.

The proposed method also replaced the laser interferometer with a high-resolution hydrophone. The method is considerably less influenced by the laser incident angle or the surface condition of the specimen than when using a laser interferometer. A hydrophone was used in this study, but if a sensor array embedded in the supporting structure replaces the hydrophone, the measurement time will be dramatically reduced.

## Figures and Tables

**Table 1:** Material properties of heat transfer model components.

	Alumina	Filled Epoxy Resin (at 20 °C)	Lead (at 20 °C)
Thermal expansion coefficient (1/K)	8 $\times \;10^{-6}$	1 $\times \;10^{-5}$	26.82 $\times \;10^{-6}$
Thermal conductivity (W/(m × K))	27	0.4252	35.37
Heat capacity at constant pressure (J/(kg × K))	900	1000	128.64
Density (kg/m^3^)	3900	1673	1138
Young’s modulus (GPa)	300	3.5	24.6
Poisson’s ratio	0.22	0.33	0.41

**Table 2:** Maximum pressure, minimum pressure, and peak-to-peak pressure of normal parts and cracked parts with various crack widths.

	Maximum Pressure (Pa)	Minimum Pressure (Pa)	Peak-to-Peak Pressure (Pa)
Normal part	2.62	−1.69	4.31
0.1 mm-wide crack (air gap)	1.86	−1.65	3.51
0.5 mm-wide crack (air gap)	1.56	−1.64	3.2
1 mm-wide crack (air gap)	0.56	−0.58	1.14

**Table 3:** ICR and the average value of the proposed laser ultrasound method and conventional water immersion ultrasound method.

Device Type	Experimental Method	Normal Part (mV)	Cracked Part (mV)	Image Contrast Ratio
Resistor	Laser ultrasonic	9.67	2.79	3.46
	Water immersion ultrasonic	8.50	6.48	1.31
Capacitor	Laser ultrasonic	2.80	1.00	2.80
	Water immersion ultrasonic	9.74	8.19	1.19

## Data Availability

Data sharing not applicable.
